# Effectiveness of a Combination of Nasturtium Herb and Horseradish Root (Angocin^®^ *Anti-Infekt N*) Compared to Antibiotics in Managing Acute and Recurrent Urinary Tract Infections: A Retrospective Real-world Cohort Study

**DOI:** 10.3390/antibiotics13111036

**Published:** 2024-11-02

**Authors:** Nina Kassner, Meinolf Wonnemann, Yvonne Ziegler, Winfried Vahlensieck, Jennifer Kranz, Karel Kostev

**Affiliations:** 1 Repha GmbH Biologische Arzneimittel, 30855 Langenhagen, Germanyyvonne.ziegler@repha.de (Y.Z.); 2 Stresemannstr, 61231 Bad Nauheim, Germany; 3 Department of Urology and Pediatric Urology, RWTH Aachen University, 52074 Aachen, Germany; jkranz@ukaachen.de; 4 Department of Urology and Kidney Transplantation, Martin-Luther-University, 06120 Halle, Germany; 5 IQVIA Epidemiology, 60549 Frankfurt, Germany

**Keywords:** Angocin^®^ *Anti-Infekt N*, herbal, antibiotic, urinary tract infection, cystitis, pyelonephritis, recurrent urinary tract infection, phytotherapy

## Abstract

Background: The goal of this study was to evaluate whether the medical recommendation of Angocin^®^ *Anti-Infekt N*, compared to standard antibiotic treatment shortly after the diagnosis of a urinary tract infection (UTI) or cystitis, is negatively associated with an early, sporadic, or recurrent UTI, subsequent antibiotic prescriptions, pyelonephritis as a renal complication, or UTI-associated sick leave. Methods: This retrospective cohort study was based on data from the IQVIA^TM^ Disease Analyzer database and included patients diagnosed with acute UTI or cystitis by physicians in Germany between 2005 and 2021, who were prescribed either Angocin^®^ or standard antibiotics within 4 days after diagnosis. Patients prescribed antibiotics were matched to those prescribed Angocin^®^ (5:1) using propensity scores. Univariable logistic and Cox regression models were used to investigate the association between Angocin^®^ prescription and the defined study outcomes. The effects of Angocin^®^ were adjusted for age, sex, insurance status, index diagnosis, and physician specialty. Results: A total of 2277 Angocin^®^ patients and 11,385 antibiotic patients were available for analysis. Compared to antibiotic prescriptions, Angocin^®^ prescription was associated with significantly lower odds of an early relapse within 1–30 days after the index date (odds ratio (OR): 0.74; 95% confidence interval (CI): 0.62–0.87; *p* < 0.001), further sporadic UTI within 31–365 days after the index date (OR: 0.68; 95% CI: 0.58–0.78; *p* < 0.001), and recurrent UTI (OR: 0.63; 95% CI: 0.48–0.82; *p* < 0.001). This was also accompanied by reduced antibiotic prescriptions (1–30 days: OR: 0.63; 95% CI: 0.53–0.74, *p* < 0.001; 31–365 days: OR: 0.56; 95% CI: 0.49–0.64, *p* < 0.001). A strong, but due to the low incidence, not significant, negative association was observed between Angocin^®^ prescription and the occurrence of pyelonephritis (hazard ratio (HR): 0.67; 95% CI: 0.43–1.06; *p* = 0.073). Conclusions: The results of this real-world data study demonstrate that Angocin^®^ can be an effective therapeutic option for managing acute and recurrent UTIs and serves as an alternative therapy to antibiotics.

## 1. Introduction

Urinary tract infections (UTIs), categorized as lower (cystitis) and upper (pyelonephritis) UTIs, affect over 150 million people globally each year [1] and are among the most common outpatient infections in Germany [2], posing a significant psycho-social burden on affected patients [3]. Various factors, including gender, genetics, behavior, and biology, influence individual susceptibility to lower UTIs [1].

By definition, UTIs are classified as uncomplicated when there are no relevant functional or anatomic abnormalities in the urinary tract, no relevant renal dysfunction, and no relevant pre-existing or concomitant diseases that promote UTI or serious complications. Uncomplicated urinary tract infections may be isolated, sporadic, or recurrent. Recent years have seen a paradigm shift in the treatment of uncomplicated UTIs. Rising antibiotic resistance among uropathogens, adverse effects on the intestinal microbiota, and the requirements of “antibiotic stewardship” have prompted a reevaluation of traditional prescription practices, which is reflected in current medical guidelines. The European Association of Urology Guidelines on Urological Infections still recommends antibiotics as the first-line treatment for uncomplicated cystitis [4]. However, the primary aim of treatment is rapid symptom relief. The updated German AWMF S3 guideline on uncomplicated urinary tract infections recommends that in non-geriatric patients, non-antibiotic therapy alone should also be considered as an alternative to antibiotic treatment [5], though the risk of pyelonephritis is slightly higher with non-antibiotic treatments (2.9% vs. 0.3%; [6]).

Several non-antibiotic pharmaceuticals are available for managing cystitis symptoms [7], including non-steroidal anti-inflammatory drugs (NSAIDs) and phytopharmaceutical preparations. Multimodal herbal preparations, especially in uncomplicated cystitis, can combat the underlying uropathogens and alleviate symptoms. Interestingly, Ehrenberg et al. reported that the prevalence of phytopharmaceuticals in UTI treatment is low among office-based physicians in Germany. However, the phytopharmaceutical preferences of physicians play an important role in prescription prevalence [8].

UTI recurrences are classified based on timing: early recurrences within two weeks suggest a relapse with the same pathogen, often due to incomplete treatment, while late recurrences beyond two weeks likely indicate new infections. Recurrent UTIs (rUTIs) are defined as more than two episodes in six months or more than three in a year, affecting one in four women.

If behavioral modifications and non-antibiotic therapies fail, low-dose antibiotics may be prescribed for rUTI prophylaxis, though compliance is often poor [9] due to adverse effects caused by microbiological collateral damage [10] on the endogenous microbiota. Furthermore, antibiotic treatment also exerts selective pressure, resulting in a significant increase in antibiotic-resistant bacteria [11]. Thus, the prevalence of antibiotic resistance in patients with uncomplicated rUTIs is thought to be relatively high [12]. Consequently, broader-spectrum antibiotics are sometimes used over the narrow-spectrum options recommended for uncomplicated UTIs [4,5,12,13].

Fortunately, there is a switch towards a more guideline-adherent prescription pattern in Germany and many other countries [14].

To possibly avoid the use of long-term antibiotic therapy in recurrent uncomplicated cystitis, alternative therapy options can be offered to premenopausal women without other comorbidities [5]. A combination of nasturtium and horseradish is mentioned as a potential herbal treatment option for rUTIs in the updated German AWMF S3 guideline on uncomplicated urinary tract infections [5].

Angocin^®^ *Anti-Infekt N* (Angocin^®^), a phytopharmaceutical with nasturtium herb (*Tropaeoli majoris herba*) and horseradish root (*Armoraciae rusticanae radix*), has been licensed in Germany for over 50 years to treat acute UTIs. The combination of the two ingredients, *Tropaeoli majoris herba* (nasturtium herb) and *Armoraciae rusticanae radix* (horseradish root), exerts a broad pharmacologic spectrum. The strong pharmacologic actions can be attributed to a combination of different isothiocyanates (ITCs, mustard oils), which are formed by enzymatic degradation of plant glucosinolates by the plant-derived enzyme myrosinase. Besides their anti-inflammatory effects, they show a broad antibacterial activity, also against multiresistant bacteria [15,16,17,18]. In addition to bacteriostatic and bactericidal effects, these ITCs can reduce bacterial motility and bacterial adhesion in vitro [19] and inhibit intraepithelial internalization processes [20] and bacterial biofilm formation by inhibiting quorum sensing as well as mature biofilms [21], making Angocin^®^ a promising option for acute and recurrent UTIs.

Previous studies support the effectiveness of Angocin^®^. In a prospective cohort study of 479 patients with UTIs, the effectiveness of Angocin^®^ therapy was found to be comparable to treatment with standard antibiotics but with significantly fewer side effects [22]. In another randomized, double-blind, placebo-controlled trial comparing Angocin^®^ with placebo, the herbal remedy significantly reduced the recurrence rate of UTI compared to placebo. The mean rate of recurrent episodes in the per protocol-treated group was 0.43 in the Angocin^®^ versus 0.77 in the placebo cohort (*p* = 0.035), without any clinically relevant differences in the reported side effects [23].

Yet, there is a lack of real-world evidence comparing Angocin^®^ to standard antibiotic therapy in lower UTIs. Given that recurrence risk, complications like pyelonephritis, and UTI symptom duration are common reasons for prescribing antibiotics, this study aimed to evaluate whether recommending Angocin^®^ shortly after UTI diagnosis is negatively associated with early, sporadic, and frequent recurrences, antibiotic prescriptions, subsequent pyelonephritis, and duration of sick leave.

## 2. Methods

### 2.1. Data Source

IQVIA, formerly Quintiles and IMS Health, Inc., is an American multinational company serving the combined industries of health information technology and clinical research. This retrospective cohort study was based on the IQVIA^TM^ Disease Analyzer database, which contains electronic medical records, including demographics, diagnoses, and prescriptions provided by office-based physicians (both GPs and specialists) in Germany. The Disease Analyzer database contains data for over 10 million patients collected between 2005 and 2021, representing eight major German regions to ensure geographical representativeness. Data acquisition and procession strictly follow German data protection laws. In Germany, the sampling methods used for the selection of physicians’ practices ensure a representative database of general and specialized practices [24]. This database has been widely used for published studies on UTIs [8,25,26].

### 2.2. Study Population

This study included patients from outpatient care settings in Germany (general, gynecological, and urological practices) with at least one of the following diagnoses according to the "**I**nternational Statistical **C**lassification of **D**iseases and Related Health Problems" ICD code between January 2005 and December 2021:

1. Acute cystitis (ICD-10: N30.0).
2. Other cystitis (ICD-10: N30.8).
3. Cystitis, unspecified (ICD-10: N30.9).
4. Urinary tract infection, site not specified (ICD-10: N39.0).

The most frequent diagnosis was N39.0, accounting for 69.4% of cases, followed by N30.9 (19.1%), N30.0 (10.8%), and N30.8 (0.7%). Patients initially diagnosed with any of these conditions were categorized into one of the two cohorts according to the **A**natomical **T**herapeutic **C**hemical (ATC) classification system:

(1) Patients with at least one prescription of Angocin^®^ on the day of diagnosis or within four days thereafter (ATC: G04BP50).

(2) Patients without Angocin^®^ prescriptions and with an antibiotic prescription (ATC: J01G1, J01E0, J01D1, J01C1, J01H1, G04A1) on the day of diagnosis or within four days thereafter.

**Figure 1:**
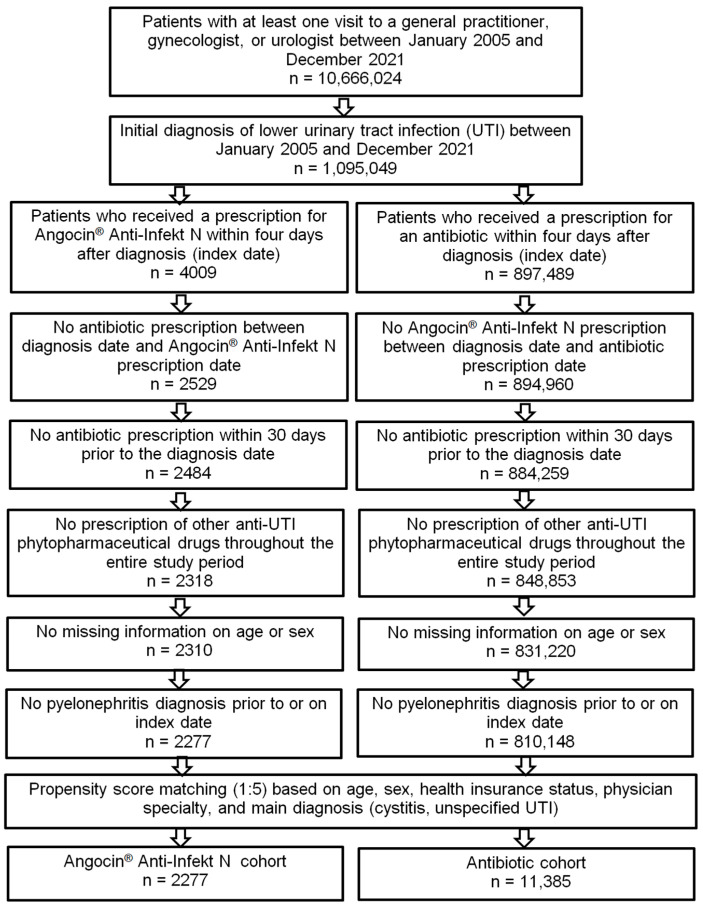
Selection of study patients.

Exclusion criteria included patients with antibiotic prescription within 30 days prior to the index date (ATC: J01, G04A1), those prescribed other herbal or synthetic medications for UTIs in the study period, those missing age and sex information, and those diagnosed with pyelonephritis prior to or on the index date (Figure 1).

### 2.3. Descriptive and Statistical Analyses

We used a matched pairs design to avoid selection bias and prevent any possible impact of co-variables on the outcomes. Patients with antibiotic prescriptions were matched to those with Angocin^®^ prescription (5:1; [27]) using a propensity score based on age, sex, health insurance status (private, statutory), index diagnosis (cystitis, unspecified UTI), and physician specialty.

Standardized mean difference (SMD) is the most frequently used measure to determine the balance of covariate distribution between treatment groups [28]. In this study, we only allowed an SMD of less than 0.1, indicating that adequate covariate balance has been achieved.

Demographic characteristics of two matched cohorts were compared using the Wilcoxon signed-rank test for continuous age and the Stuart–Maxwell and McNemar tests for categorical variables.

Univariable logistic regression models were used to investigate the association between Angocin^®^ prescription and the risk of an early recurrence diagnosis within 1–30 days, a sporadic recurrence within 31–365 days after diagnosis, the risk of rUTI and the risk of at least one antibiotic prescription in the period 1–30 days after and 31–365 days after the index date in comparison to antibiotic therapy. rUTIs were defined as at least three diagnoses of UTIs in the period 1–365 days after or at least two diagnoses of UTIs in the period 1–183 days after the index date.

The differences in percentages of patients with initial documentation of pyelonephritis (ICD 10: N10–12) following the diagnosis of a UTI between the Angocin^®^ and the antibiotic cohort were estimated using Kaplan–Meier curves. Incidence rates in cases per 1000 person-years were calculated. Cox regression models were used to investigate the association between Angocin^®^ prescription and risk of pyelonephritis up to three years after the index date. Essentially, a hazard is the event rate for a particular group of patients, and the hazard ratio is a quotient of the hazards of two groups and states, measuring how much higher the event rate is in one group than in the other.

All regression models (logistic or Cox) were run separately for men and women as well as for age groups (<30 years, 31–45 years, 46–60 years, >60 years). In all analyses, a *p*-value of <0.05 was considered statistically significant. Analyses were conducted using SAS Vers. 9.4 (SAS Institute, Cary, NC, USA).

## 3. Results

### 3.1. Baseline Characteristics of Study Patients

Of the 1,095,049 patients diagnosed with acute cystitis (ICD-10: N30.0), other cystitis (ICD-10: N30.8), unspecified cystitis (ICD-10: N30.9), or urinary tract infection, site not specified (ICD-10: N39.0) and having an observation time of at least 365 days prior to the index date, either Angocin^®^ was prescribed to 4009 (0.37%) and antibiotics to 897,489 (81.96%) within four days after diagnosis. A total of 193,551 patients (17.68%) received other therapies. Patients with combination therapies, i.e., Angocin^®^ and antibiotic prescription, were excluded from both groups. This reduced the groups down to 2529 patients (0.23%) and 894,960 patients (81.96%). The following patients were also excluded in case of an antibiotic prescription 30 days prior to the date of diagnosis and in case of prescribing a combination therapy with other UTI drugs (e.g., NSAIDs, mannose-, methionine-, arbutin-containing drugs, or other phytopharmaceuticals). This resulted in 2318 Angocin^®^ patients (0.21%) and 848,853 antibiotic patients (77.52%). Finally, all patients with either missing information on age or sex or with a pyelonephritis diagnosis prior to or on the diagnosis date were excluded. This resulted in a final number of 2277 Angocin^®^ (0.21%) and 810,148 antibiotic patients (73.98%) prior to propensity score matching. After 1:5 propensity score matching, 2277 Angocin^®^ patients and 11,385 antibiotic patients were available for analysis (Figure 1).

Table 1 shows the baseline characteristics of the study patients. Prior to matching, Angocin^®^ patients were significantly younger, had a higher proportion of private health insurance, and were more frequently treated by urologists compared to those prescribed antibiotics. After matching, no significant differences were observed between the two study cohorts in terms of age (mean 45.7 years), sex (83.9% female), private health insurance coverage (19.8% private), diagnosis of cystitis (ICD-10: N30.0, N30.8, N30.9 = 31.4%), or diagnosis by GP (41.6%), urologist (48.4%) or gynecologist (10.0%).

### 3.2. Early and Sporadic UTI

**Figure 2:**
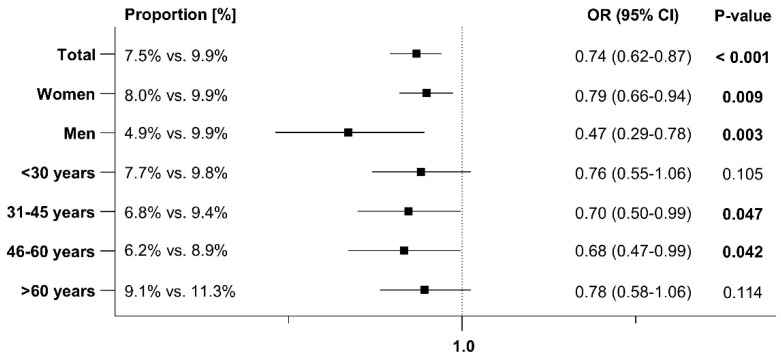
Association between Angocin^®^ prescription and further confirmed diagnosis of early UTI within 1–30 days after the index date [Angocin^®^ versus antibiotics].

**Figure 3:**
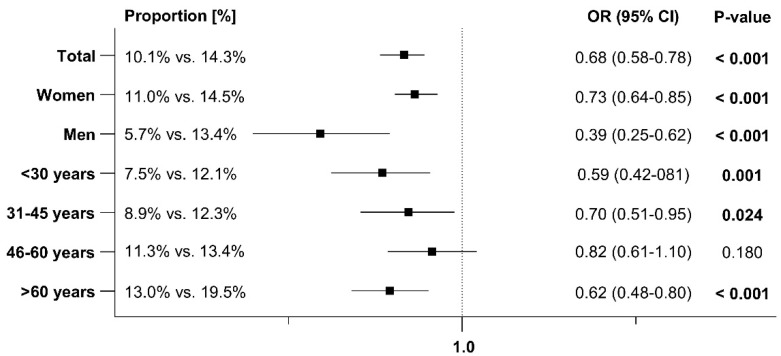
Association between Angocin^®^ prescription and further confirmed diagnosis of sporadic UTI 31–365 days after the index date [Angocin^®^ versus antibiotics].

An early recurrence within 1–30 days after the index date was documented in 7.5% of patients prescribed Angocin^®^ compared to 9.9% of those prescribed antibiotics. A sporadic recurrence within 31–365 days after the index date was observed in 10.1% of Angocin^®^ patients versus 14.3% of antibiotic patients. The results of the univariable logistic regression model are displayed in Figure 2 and Figure 3. Angocin^®^ prescription was associated with significantly lower odds of a further confirmed early and sporadic UTI diagnosis (odds ratio [OR]: 0.74; 95% confidence interval [CI]: 0.62–0.87 within 1–30 days and OR: 0.68; 95% CI: 0.58–0.78 within 31–365 days after the index date). This association was significant in both men and women and was consistent across various age groups, although not significant in all age groups. The association was stronger in men than in women (Figure 2 and Figure 3).

### 3.3. Recurrent Urinary Tract Infections (rUTI)

**Figure 4:**
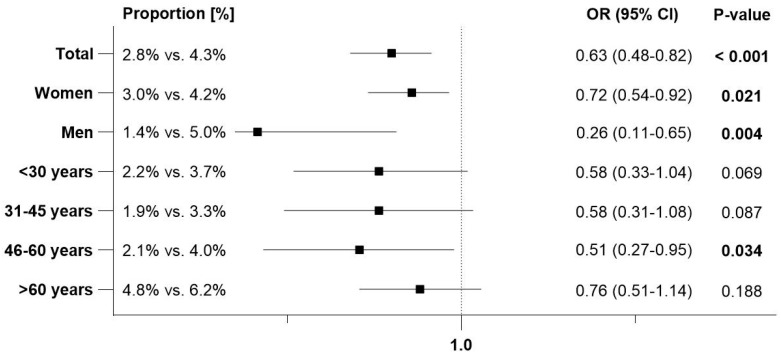
Association between Angocin^®^ prescription and probability of rUTI [Angocin^®^ versus antibiotics].

rUTIs, defined as at least three UTI diagnoses within 1–365 days or at least two diagnoses within 1–183 days after the index date, were documented in 2.8% of patients with Angocin^®^ prescriptions and 4.3% of patients with an antibiotic prescription. The results of the univariable logistic regression model are displayed in Figure 4. Angocin^®^ prescription was associated with significantly lower odds of rUTIs (OR: 0.63; 95% CI: 0.48–0.82). This association was significant in both men and women, respectively, with a stronger effect observed in men. Among patients aged >60 years, the association was negative but not statistically significant (Figure 4).

### 3.4. Antibiotic Prescriptions After the Index Date

**Figure 5:**
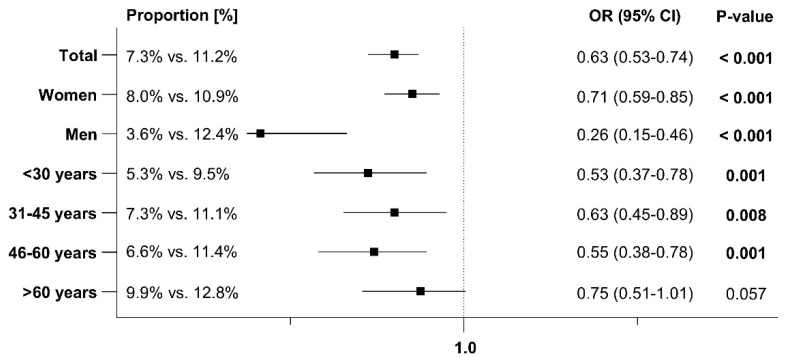
Association between Angocin^®^ and antibiotic prescription and the probability of a further antibiotic prescription 1–30 days after the index date [Angocin^®^ versus antibiotics].

**Figure 6:**
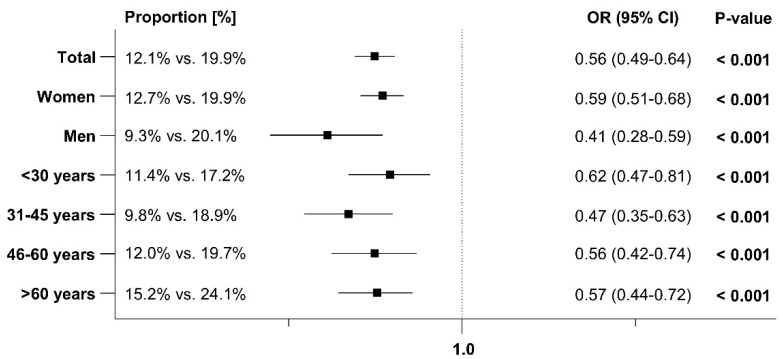
Association between Angocin^®^ and antibiotic prescription and the probability of a further antibiotic prescription 31–365 days after the index date [Angocin^®^ versus antibiotics].

At least one additional antibiotic prescription for treating an early recurrence was issued to 7.3% of patients with Angocin^®^ prescriptions and 11.2% of patients with antibiotic prescriptions within 1–30 days after the index date. Within 31–365 days after the index date, 12.1% of Angocin^®^ patients and 19.9% of antibiotic patients received at least one further antibiotic prescription. The results of the univariate logistic regression model are displayed in Figure 5 and Figure 6. Angocin^®^ prescription was associated with significantly lower odds of further antibiotic prescriptions in both time periods (1–30 days after the index date: OR: 0.63; 95% CI: 0.53–0.74, 31–365 days after the index date: OR: 0.56; 95% CI: 0.49–0.64). This association was observed across all subgroups, with a stronger effect in men than in women (Figure 5 and Figure 6).

### 3.5. Sick Leave After the Index Date

Overall, 4.1% of patients prescribed Angocin^®^ and 4.0% of those prescribed antibiotics took at least three days of sick leave due to UTI after beginning therapy (sick leave on the day of prescription was excluded). There was no significant association between Angocin^®^ prescription and the odds of taking sick leave (OR: 1.03; 95% CI: 0.80–1.34).

### 3.6. Incidence of Pyelonephritis

**Figure 7:**
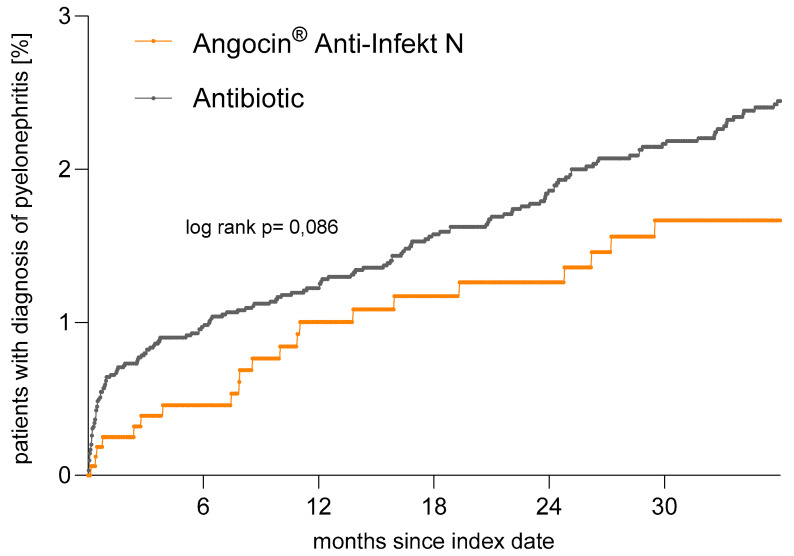
Time to pyelonephritis diagnosis in patients with Angocin^®^ and antibiotic prescriptions (Kaplan–Meier curves).

Pyelonephritis is a relatively rare event, occurring within 3 years after the index date (Figure 7, Table 2). The incidence of pyelonephritis was 6.2 cases per 1000 person-years among Angocin^®^ patients and 9.2 cases per 1000 person-years among antibiotic patients. Although both Kaplan–Meier curves and regression analyses show a clear negative association between Angocin^®^ prescription and the incidence of pyelonephritis, this association did not reach statistical significance based on the relatively rare nature of the related events (hazard ratio (HR): 0.67; 95% CI: 0.43–1.06).

Moreover, if the analysis is restricted to recommended first-choice antibiotics for the treatment of uncomplicated UTI (German S3 guideline [5]), the negative association is markedly reduced. This suggests that pyelonephritis occurs less frequently with specific antibiotic therapies recommended by the guideline for uncomplicated UTIs.

## 4. Strengths and Limitations

The major strengths of this study include its large sample size and the inclusion of data from different physician specialties. However, several relevant limitations should be noted. First, the number of patients treated with Angocin^®^ was markedly lower than those treated with antibiotics, likely due to Angocin^®^’s over-the-counter (OTC) status and its lower prescription rate compared to antibiotics, which are prescription-only (Rx). The database only includes data on herbal medicines prescribed by physicians, so the use of additional OTC medications by patients cannot be ruled out. Self-medication, in addition to prescribed treatments, plays a significant role in the German healthcare system [29]. The comparison with antibiotics may be questioned, as patients receiving antibiotics on the day of diagnosis might have been more severely ill than those treated with phytodrugs like Angocin^®^ and other OTC medications. However, this assumption can be challenged; a study by Ehrenberg et al. found that a practice’s preference for phytopharmaceuticals was associated with a six-fold increase in phytodrug prescriptions, independent of diagnosis and patient characteristics [8].

Second, UTI diagnoses were assessed based on ICD codes entered by GPs, urologists, and gynecologists, which do not differentiate between complicated and uncomplicated UTIs, nor do they provide information on urine culture tests that identify causative bacteria. Additionally, since 2017, asymptomatic bacteriuria is no longer considered to require treatment.

Lastly, data on socioeconomic status and lifestyle-related risk factors were not available. Despite these limitations, the German IMS^®^ database used in this study has been validated in numerous medical studies, supporting the credibility of our findings. To reduce any potential bias, regression models were applied separately for three age groups and for men and women, in addition to the adjustments included in the regression models.

## 5. Discussion

The aim of the present study was to investigate the association between Angocin^®^ prescription and the occurrence of UTIs (early, sporadic, and recurrent) following treatment, subsequent antibiotic prescription, pyelonephritis diagnosis, and sick leave compared to antibiotic treatment. This large retrospective cohort study provides valuable insights into the potential of Angocin^®^ for managing acute and recurrent UTI episodes and highlights its potential role in reducing antibiotic use and combating antibiotic resistance.

A key finding of our study is that Angocin^®^ prescription was associated with lower incidences of early, sporadic, and recurrent UTI diagnosis compared to antibiotic therapy. These results suggest that Angocin^®^ may effectively reduce the recurrence rate of UTIs, a common challenge in managing this condition. The overall OR for the association between Angocin^®^ and rUTI was 0.63, indicating a significant reduction of rUTI incidence compared to antibiotic therapy.

These data support the use of Angocin^®^ as a non-antibiotic treatment option for acute and rUTIs and are in line with findings from previous clinical studies. Angocin^®^ therapy leads to a significant reduction of subsequent antibiotic prescriptions, potentially lowering the risk of gut microbiota disruption and further collateral damages, adverse effects, and the development of antibiotic resistance.

Angocin^®^ has been approved for use in acute UTIs in Germany since 2005 and is currently mentioned in the updated German AWMF S3-guideline as a potential option for uncomplicated rUTIs [5]. Evidence generated from the present real-world data analysis further supports the previous findings and recommendations.

The efficacy and safety of Angocin^®^ in treating acute and recurrent UTIs have been demonstrated in several clinical trials and prospective cohort studies involving both adults and children [22,23,30]. In a randomized, double-blind, placebo-controlled trial, Angocin^®^ significantly reduced the recurrence rate compared to placebo (0.43 vs. 0.77, *p* = 0.035) in the per-protocol analysis [23].

Angocin^®^ contains horseradish root and nasturtium herb as active ingredients. The strong pharmacological effects (antibacterial, anti-inflammatory, anti-biofilm, anti-adhesive, anti-internalization) can be ascribed to their active ingredients, the isothiocyanates (ITCs) [15,16,17,18,19,20,21]. As ITCs are primarily excreted via the kidneys, high local concentrations achieved in the urine suggest their clinical effectiveness against UTIs [31]. In addition to reducing the incidence of early, sporadic, and recurrent UTIs, Angocin^®^ prescription was associated with a lower likelihood of subsequent antibiotic use. This finding is particularly significant for antibiotic stewardship and the global effort to combat antibiotic overuse and resistance. The reduced need for antibiotics with Angocin^®^ underscores its potential to minimize antibiotic use, which is crucial from a public health perspective. Additionally, no negative association was observed between the duration of UTI-related sick leave among patients treated with Angocin^®^ compared to those prescribed antibiotics.

It is important to note that negative associations between Angocin^®^ prescriptions and the occurrence of early, sporadic, and recurrent UTIs, as well as subsequent antibiotic prescriptions, were consistent across different demographic groups, including men and women. While women are more prone to UTIs due to anatomical and other factors, men experience similar symptom severity and face the same risk of urinary complications, such as pyelonephritis, underscoring the need for effective treatment [32,33]. In our study, the effectiveness of Angocin^®^ was stronger in men than in women, suggesting a potential sex-specific benefit in UTI management.

This study also aimed to assess the occurrence of pyelonephritis within three years following the index date and its potential association with Angocin^®^ use compared to antibiotics. Pyelonephritis, a severe upper UTI, can lead to substantial morbidity and healthcare resource utilization, making its prevention and management a matter of paramount importance [34,35]. This serious complication typically occurs in about 0.3% of lower UTI cases, with higher rates reported among patients receiving non-antibiotic symptomatic treatments [6].

However, this could not be confirmed in this study for the treatment with Angocin^®^. Our findings reveal that pyelonephritis was infrequent within the three-year follow-up period. While both Kaplan–Meier survival curves and regression analyses illustrated a clear trend towards a lower incidence of pyelonephritis in the Angocin^®^ group compared to the antibiotic group, this association did not reach statistical significance.

The infrequent occurrence of pyelonephritis within the study population limited the statistical power to detect significant differences between the treatment groups, introducing variability that made it difficult to achieve statistical significance.

Despite this, the observed trend towards a reduced incidence of pyelonephritis in the Angocin^®^ group is noteworthy and aligns with the broad pharmacological effects of its active ingredients, nasturtium and horseradish, known for their antibacterial and anti-inflammatory properties in the urinary tract. Given the severity of pyelonephritis and its potential complications, even a trend toward risk reduction is clinically relevant.

However, when the analysis is restricted to first-choice antibiotics recommended for the treatment of uncomplicated UTI (German S3 guideline) [5], the negative association is less pronounced. This indicates that pyelonephritis occurs less frequently with certain guideline-recommended antibiotic therapies. This aligns with the fact that first-choice antibiotics have resistance rates of less than 20% against *E. coli*, an acceptable rate for empirical use, leading to fewer treatment failures [5]. Overall, these findings support the use of Angocin^®^ as a non-antibiotic symptomatic and antibacterial treatment for acute and recurrent UTIs, consistent with previous clinical studies. Angocin^®^ is well-tolerated, significantly reduces antibiotic use, and helps preserve gut microbiota, preventing collateral damage and reducing the risk of antibiotic resistance.

## 6. Conclusions

The results of this study suggest that Angocin^®^ may be a valuable therapeutic option for managing acute episodes of UTIs as a monotherapy. Its association with a reduced incidence of early, sporadic, and recurrent UTIs, lower antibiotic use, and decreased risk of pyelonephritis underscores its potential as an alternative to antibiotics. Further research, including prospective clinical trials, is warranted to validate these findings.

## Figures and Tables

**Table 1:** Basic characteristics of study patients.

	Prior to Matching			After Matching		
Variable	Patients with Angocin^®^ Prescription	Patients with Antibiotic Prescription	*p*-Value	Patients with Angocin^®^ Prescription	Patients with Antibiotic Prescription	*p*-Value
*n*	2277	810,148		2277	11,385	
Age (mean, SD)	45.7 (19.8)	49.8 (21.6)	<0.001	45.7 (19.8)	45.7 (19.7)	0.985
<30 years (*n*, %)	587 (25.8)	187,446 (23.1)	<0.001	587 (25.8)	2995 (26.3)	0.946
31–45 years (*n*, %)	572 (25.1)	166,550 (20.6)		572 (25.1)	2827 (24.8)	
46–60 years (*n*, %)	533 (23.4)	171,438 (21.2)		533 (23.4)	2679 (23.5)	
>60 years (*n*, %)	585 (25.7)	284,714 (35.1)		585 (25.7)	2884 (25.3)	
Sex: female (%)	1911 (83.9)	647,128 (79.9)	<0.001	1911 (83.9)	9555 (83.9)	1.000
Private health insurance coverage (*n*, %)	450 (19.8)	69,625 (8.6)	<0.001	450 (19.8)	2250 (19.8)	1.000
Cystitis (*n*, %)	715 (31.4)	263,374 (32.5)	<0.001	715 (31.4)	3575 (31.4)	1.000
Therapy by general practitioner (*n*, %)	947 (41.6)	540,168 (66.7)	<0.001	947 (41.6)	4735 (41.6)	1.000
Therapy by gynecologist (*n*, %)	227 (10.0)	123,139 (15.2)		227 (10.0)	1135 (10.0)	
Therapy by urologist (*n*, %)	1103 (48.4)	146,841 (18.1)		1103 (48.4)	5515 (48.4)	

**Table 2:** Association between Angocin^®^ prescription and probability of pyelonephritis [Angocin^®^ versus antibiotic].

Patient Group	Events per 1000 Person-Years in Patients with Angocin^®^ Prescription (%)	Events per 1000 Person-Years in Patients with Antibiotic Prescription (%)	Hazard Ratio (95% CI)	*p*-Value
Total	6.2	9.2	0.67 (0.43–1.06)	0.073
Women	7.2	10.2	0.70 (0.44–1.11)	0.125
Men	1.7	4.1	0.42 (0.06–3.21)	0.403
Age groups				
<30 years	7.4	17.6	0.42 (0.17–1.04)	0.060
31–45 years	7.8	9.8	0.76 (0.33–1.79)	0.531
46–60 years	5.4	6.6	0.82 (0.32–2.09)	0.670
>60 years	5.1	5.0	1.02 (0.39–2.66)	0.966

## Data Availability

The data presented in this study are available on request from the corresponding author.
